# Favipiravir in early symptomatic COVID-19, a randomised placebo-controlled trial

**DOI:** 10.1016/j.eclinm.2022.101703

**Published:** 2022-10-20

**Authors:** James H. McMahon, Jillian S.Y. Lau, Anna Coldham, Janine Roney, Michelle Hagenauer, Sally Price, Mellissa Bryant, Jill Garlick, Anne Paterson, Sue J. Lee, Jess O'Bryan, Anna Hearps, Gilda Tachedjian, Henry Pinskier, Cameron Phillips, Stuart Garrow, Nathan Pinskier, Robert Melvin, Luke Blakeway, Jessica A. Wisniewski, Sally Byers, Gnei Z. Badoordeen, Stephanie Pereira, Katherine Pragastis, Jason A. Trubiano, Kyra Y.L. Chua, Marion Kainer, James S. Molton, Bradley J. Gardiner, Anna B. Pierce, Allen Cheng, Benjamin A. Rogers, Anton Y. Peleg

**Affiliations:** a Department of Infectious Diseases, Alfred Hospital and Central Clinical School, Monash University, Melbourne, Australia; b Department of Infectious Diseases, Monash Medical Centre, Melbourne, Australia; c Department of Infectious Diseases, Eastern Health, Melbourne, Australia; d Life Sciences Discipline, Burnet Institute, Melbourne, Australia; e Department of Microbiology, Monash University, Clayton, Australia; f Department of Microbiology and Immunology at the Peter Doherty Institute for Infection and Immunity, University of Melbourne, Australia; g Onsite Doctor and OSD.Care, Melbourne, Australia; h Department of Emergency Medicine, Alfred Hospital, Melbourne, Australia; i Department of Infectious Diseases, Austin Hospital, Melbourne, Australia & Department of Medicine, University of Melbourne; j Department of Infectious Diseases, Western Health, Melbourne, Australia; k Epworth Healthcare, Melbourne, Australia; l South East Public Health Unit, Monash Health, Melbourne, Australia; m Monash University School of Clinical Sciences at Monash Health, Clayton, Australia; n Infection Theme, Monash Biomedicine Discovery Institute, Department of Microbiology, Monash University, Melbourne, Australia

**Keywords:** Favipiravir, COVID-19, Randomised clinical trial

## Abstract

**Background:**

Well tolerated antivirals administered early in the course of COVID-19 infection when the viremia is highest could prevent progression to severe disease. Favipiravir inhibits SARS-CoV-2 viral replication *in vitro* with evidence of clinical benefit in open label trials. Placebo controlled studies of people with early symptomatic COVID-19 with regular assessments of SARS-CoV-2 viral load can determine if it has an antiviral effect and improves clinical outcomes.

**Methods:**

People with PCR-confirmed COVID-19 and 5 days or less of symptoms were randomised 1:1 to favipiravir 1800 mg on day 1, then 800 mg twice daily or matched placebo for 14 days. SARS-CoV-2 viral load was quantitated from second daily self-collected nose-throat swabs while receiving study drug. The primary endpoint was time to virological cure defined as 2 successive swabs negative for SARS-CoV-2 by PCR and secondary outcomes were progression of disease severity, symptom resolution and safety.

**Findings:**

Between 31 July 2020 and 19 September 2021, 200 people were enrolled (199 in the community, 1 in hospital) with 190 receiving one or more doses of drug (modified intention to treat [mITT] population). There was no difference in time to virological cure (Log-rank *p*=0.6 comparing Kaplan Meier curves), progression to hospitalisation (14 favipiravir, 9 placebo; *p*=0.38), time to symptom resolution (cough, fever, sore throat) and there were no deaths. 51 people related an adverse event that was possibly drug related, but these were evenly distributed (*n*=24 favipiravir, *n*=27 placebo). Sensitivity analyses where the definition of virological cure was changed to: a single negative PCR, exclude datapoints based on the presence or absence of human DNA in the swab, a SARS-CoV-2 viral load < 300 copies/mL being considered negative all demonstrated no difference between arms.

**Interpretation:**

Favipiravir does not improve the time to virological cure or clinical outcomes and shows no evidence of an antiviral effect when treating early symptomatic COVID-19 infection.

**Funding:**

The study was supported in part by grants from the Commonwealth Bank Australia, the Lord Mayor's Charitable Foundation, Melbourne Australia and the Orloff Family Charitable Trust, Melbourne, Australia. JHM is supported by the Medical Research Future Fund, AYP, JT are supported by the Australian National Health and Medical Research Council.

**Research in context**

**Evidence before this study**

Prior to this trial there were reports from in vitro studies and open label clinical trials where favipiravir conferred clinical benefit in people hospitalised with COVID-19. In addition, SARS-CoV-2 infection has a period of high viremia during the first week of illness with progression to hospitalisation, oxygen requirement and death in the second week when viremia is decreasing. Favipiravir is a small molecule antiviral that interferes with viral replication that is safe when used to treat other viral infections such as influenza. It therefore warranted investigation in a placebo controlled trial of early COVID-19 to understand if it had an antiviral effect and prevented progression to severe disease in people with symptomatic COVID-19.

**Added value of this study**

This trial combined daily high-dose (2000 mg) Disulfiram for 28 days with standard dosing of vorinostat on days 8–10 and 22–24. There was evidence of latency reversal in the first 2 participants enrolled but they both experienced severe neurotoxicity and the trial was suspended. The clinical presentations were consistent with prior descriptions of disulfiram toxicity but as plasma concentrations of Disulfiram were low an unexplained interaction between disulfiram and vorinostat cannot be excluded.

**Implications of all the available evidence**

Due to the severity of the neurotoxicity observed, the combination of daily high-dose (2000 mg) Disulfiram and standard dosing (400 mg) of Vorinostat should not be pursued in clinical trials targeting persistent HIV. Alternative and shorter regimens combining Vorinostat and Disulfiram could only be considered in a clinical trial with carefully designed dose escalation.

Alt-text: Unlabelled box

## Introduction

The COVID-19 pandemic has led to over half a billion infections and 6 million deaths by mid 2022. Treatments that are safe and prevent severe outcomes such as death and the need for hospitalisation have been a global priority. Therapies initially approved and recommended for the treatment of COVID-19 were potent anti-inflammatories such as dexamethasone and baricitinib that decreased mortality for people already diagnosed with severe COVID-19 receiving oxygen therapy as hospital inpatients.^1^^,^^2^

The natural history of SARS-CoV-2 infection involves a period of high viremia during the first week. During the second week, as the viraemia clears, some individuals are at risk of pneumonia and multi organ involvement which can result in hospitalisation, oxygen requirement and death.^3^ This later phase of the illness, associated with high levels of inflammation, is where potent anti-inflammatories may provide benefit. There has been increased interest in antivirals that can be safely administered early in the course of infection as outpatients to decrease the viral burden and prevent progression to severe disease. Therapies in this category have recently been approved but have limitations including the need for intravenous administration, and drug–drug interactions.^4^, ^5^, ^6^

Favipiravir, is a small molecule antiviral which in its active form interferes with viral replication by competing with purine nucleosides for incorporation into the nascent viral RNA and inhibiting viral RNA dependent RNA polymerase activity.^7^ Due to its mechanism of action, activity against SARS-CoV-2 *in vitro*,^8^ and known safety profile when treating infections such as influenza, favipiravir has been studied in many clinical trials in both an inpatient and outpatient setting for COVID-19. This includes early reports from open-label studies in China showing improved clinical recovery and viral clearance.^9^^,^^10^

These *in vitro* and early clinical reports combined with oral dosing, its safety profile, and an understanding that antivirals would be most beneficial early in the course of infection, provided a strong rationale for studying this agent in COVID-19 infection. To avoid bias inherent in open label designs and to rigorously assess for an antiviral effect, we performed a placebo-controlled trial with regular quantitation of SARS-CoV-2 viral load.

## Methods

### Study design

We performed a randomised placebo-controlled phase 2 trial of favipiravir versus matched placebo (NCT04445467) in individuals infected with COVID-19.^11^ The study was approved by the Alfred Ethics Committee (No 406/20) and all participants provided informed consent. The trial was overseen by an independent safety monitoring committee (SMC). Eligible participants with confirmed COVID-19 were randomised 1:1 to favipiravir or placebo for 14 days in addition to standard of care. Participants were recruited from July 2020 to September 2021 and were followed for a minimum of 28 days. Participants could be either in the community or a hospital inpatient at time of recruitment.

The sample size calculation was based on estimates from two studies available prior to the trial commencing.^9^^,^^10^ One randomised trial of 236 patients reported 61% of people receiving favipiravir compared to 52% of people receiving umifenovir met the primary clinical recovery endpoint by day 7.^10^ In a non-randomised study of 80 people (subsequently retracted), 90% of the favipiravir group achieved viral clearance compared to 57% of those on lopinavir/ritonavir, with a significantly faster rate of clearance in patients who received favipiravir (Hazard Ratio (HR) 3.43, 95% CI 1.16 to 10.1).^9^ We assumed 80% of patients on favipiravir and 60% of patients in the placebo arm would reach virological cure with twice as fast a rate of cure occurring in the favipiravir arm (HR 2.0). Assuming an alpha of 0.05, 86 participants in each arm would allow a log rank comparison with 80% power. Allowing for 10% lost to follow-up, we aimed to recruit 190 participants.

Favipiravir dosing was based on a 50% effective concentration or EC_50_ of 61.88 μM (9.7 μg/ml) *in vitro*^8^ and the knowledge that mean daily trough levels over 20 μg/ml are achieved with the same dosing proposed in this trial.^12^ Participants were given 1800 mg of favipiravir twice daily on Day 1 followed by 800 mg twice daily or identical placebo tablets. Duration of dosing was 14 days based on reports from available published trials at study initiation.^9^^,^^10^

### Patient population

We enrolled adults (≥18 years old) with PCR confirmed COVID-19 on nasopharyngeal or combined nose and throat swab and onset of COVID-19 related symptoms (one or more of: fever, cough, sore throat, shortness of breath, fatigue, myalgia) in the prior 5 days. Participants were excluded if they were enrolled into another COVID-19 antiviral treatment trial, or were pregnant or breastfeeding. Female participants of childbearing potential were required to have a negative pregnancy test prior to enrolment. Individuals with chronic liver disease (Child-Pugh C) or renal impairment requiring dialysis were also excluded. Recruitment was predominately via community enrolment through a referral network of hospitals, a quarantine facility for international arrivals and COVID-19 testing centres across metropolitan Melbourne. Inpatient recruitment was through three tertiary referral hospitals caring for patients with COVID-19 (The Alfred Hospital, Monash Health and Austin Health).

### Study endpoints

The primary study endpoint was time to virological cure as defined by 2 successive throat (or combined nose/throat) swabs negative for SARS-CoV-2 by PCR. Secondary endpoints included safety, defined as all adverse events possibly related to study treatment, and time from randomisation to a two point improvement (from the status at randomisation) on a 7-point ordinal scale. The ordinal scale consisted of the following categories: 1, not hospitalised with resumption of normal activities; 2, not hospitalised, but unable to resume normal activities; 3, hospitalised, not requiring supplemental oxygen; 4, hospitalised, requiring supplemental oxygen; 5, hospitalised, requiring nasal high-flow oxygen therapy, non-invasive mechanical ventilation, or both; 6, hospitalised, requiring ECMO, invasive mechanical ventilation, or both; and 7, death. Due to the requirement to recruit people within 5 days of symptom onset and therefore enrolment in the community prevention of progression along this 7-point scale, including prevention of hospitalisation, was assessed. Time from randomisation to resolution of symptoms and change in SARS-CoV-2 viral load from nose/throat swabs over time were also examined. Adverse events were captured and included any new symptom that occurred after at least one dose of study drug in the modified intention to treat (mITT) population. Monitoring of standard blood parameters (full blood exam, liver and renal function) at baseline and day 28 occurred when the study was initiated but the protocol was amended to waive laboratory monitoring after an interim blinded analysis of the first 82 participants showed no difference in blood parameters by treatment arm.

### Randomisation

Randomisation was performed at the Alfred Hospital Clinical Trials Pharmacy using computer generated block-randomisation lists with a block size of 6 and a 1:1 allocation ratio of favipiravir to placebo. Randomisation was stratified by study site. All participants enrolled in the community were considered as a single study site.

### Study assessments

After assessing eligibility and obtaining informed consent, participants were provided with their study medication and baseline blood was sampled along with a self-collected combined nose and throat swab to assess SARS-CoV-2 viral load. Clinical review was conducted by telephone for symptoms and adverse effects, and repeat self-collected swabs were performed every second day until day 14, the last day of study drug. Participants were provided with video and paper leaflet instructions on how to perform self-collected swabs for SARS-CoV-2 testing according to Australian national guidelines and were supervised by study staff in person or on the phone as necessary. Once collected, swabs were stored in the participants’ domestic refrigerator, collected by study staff within 24 h and transported back to the Alfred hospital for processing of viral transport medium (VTM) into aliquots and storage at −80 °C. Clinical review and safety bloods were performed 28 days following randomisation.

SARS-CoV-2 viral load was quantitated from aliquots of VTM from participant swabs using reverse transcription-quantitative PCR (RT-qPCR). In short, viral RNA was extracted from 200 μL VTM using the Direct-zol RNA miniprep kit (Zymo Research, Irvine, CA). The nucleocapsid N1 SARS-CoV-2 target and a human control gene Ribonuclease P (RNaseP) were each amplified in duplicate using published primer and probe sequences (Integrated DNA Technologies, Coralville, IA).^13^ Internal controls including positive template and no-template controls as well as extraction controls were included to confirm assay performance. Viral load in copies/mL of VTM was determined by extrapolation from a standard curve generated from serial dilution of control DNA plasmid containing the target gene (Integrated DNA Technologies) and adjusted for volume of VTM the sample was eluted from.

Adverse events were assessed by study investigators and any events assessed as possibly, probably, or definitely related to study drug were reported as related to study drug. Serious adverse events (hospitalisation, death, disability, or other medically significant event as determined by the investigator) were also assessed for relatedness.

### Study population and statistical analysis

All participants who consented and were randomised made up the intent-to-treat (ITT) population. The modified intent-to-treat (mITT) consisted of all participants who received at least one dose of study drug and had a SARS-CoV-2 nasopharyngeal PCR. The per protocol (PP) population consisted of all mITT participants who adhered to study procedures including receipt of study drug for 14 days. Comparisons were made using a chi-squared test for categorical outcomes. Time to virological cure and other time-to event outcomes (resolution of clinical symptoms, progression to hospitalisation) were compared between the favipiravir and placebo arms with the log-rank test using a Kaplan-Meier time to event analysis.

Assessments of virological cure were done in multiple ways. For the primary endpoint analysis samples were excluded if both SARS-CoV-2 RNA and the control gene were undetectable. Additional sensitivity analyses were performed for this endpoint by considering low detectable results for SARS-CoV-2 RNA (< 300 copies/mL) as undetectable, by disregarding results for the control gene and only analysing based on results of SARS-CoV-2 RNA, excluding the result if the control gene was undetectable regardless of the results for SARS-CoV-2 RNA and by defining clearance based on a single SARS-CoV-2 RNA as undetectable.

### Role of the funding source

The study was supported in part by grants from the Commonwealth Bank Australia, the Lord Mayor's Charitable Foundation, Melbourne Australia and the Orloff Family Charitable Trust, Melbourne, Australia. Funders had no role in data collection, analysis, or interpretation; trial design; patient recruitment; the writing of the manuscript or the decision to submit it for publication

None of the authors were paid to write this article by a pharmaceutical company or other agency. All authors were not precluded from accessing data in the study, and they accept responsibility to submit for publication.

## Results

**Figure 1:**
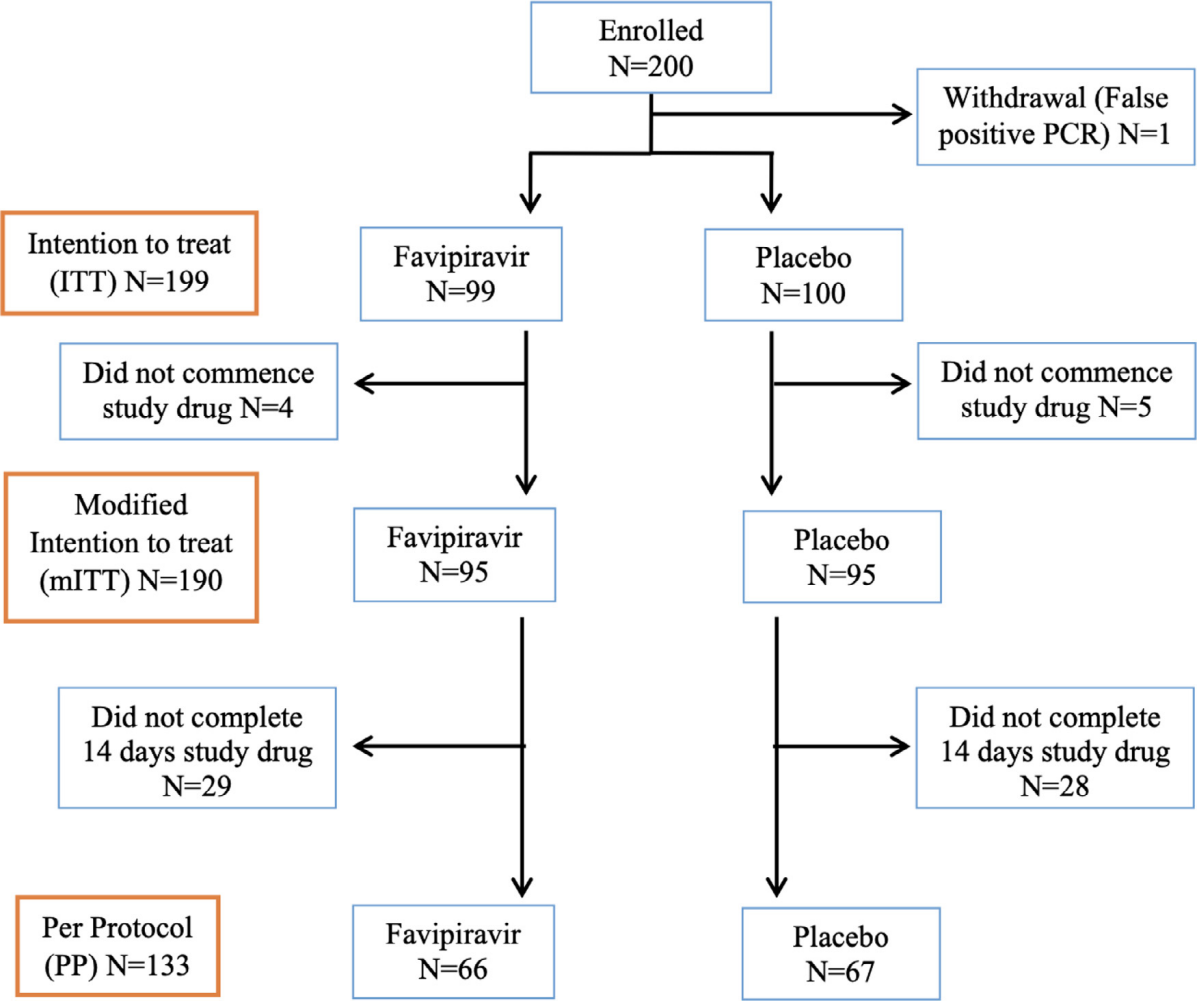
**CONSORT Flow diagram**. Defined study populations listed on left of chart: Intention to treat (ITT) enrolled and met study inclusion criteria**,** modified intention to treat (mITT) as per ITT and commenced study drug, per protocol as per mITT and completed 14 days study drug.

The trial enrolled 200 participants between 31 July 2020 and 19 September 2021 with all but one participant being enrolled in the community setting. One participant was subsequently found to be a false positive diagnosis as their diagnostic test was involved in a laboratory contamination event and was withdrawn from the trial leaving 199 participants in the intention to treat population. The mITT population comprised 190 people with 9 people withdrawing before commencing study drug (4 randomised to favipiravir and 5 to placebo),. 133 people completed 14 days of study drug as per protocol (66 randomised to favipiravir and 67 to placebo) (Figure 1). Participants did not have their viral isolates sequenced to determine their viral variant but the highest periods of enrolment were during periods of community transmission of the Wuhan ancestral strain in 2020 and the Delta variant in 2021

Baseline demographic and clinical characteristics were similar between the two groups (Table 1). Median age was 36.0 years, 68.8% identified as Caucasian/White and 12.6% as Asian. The most common risk factor for acquisition was contact with someone known to be infected with COVID-19 (67.3%) and 66.2% of people were categorised as not hospitalised but unable to resume their normal activities at enrolment (WHO category 2).

**Table 1:** Baseline characteristics.

	Overall (*n*=199)	Favipiravir (*n*=99)	Placebo (*n*=100)
**Demographics**			
Median age (IQR)	36.0 (28.0, 51.0)	36.0 (28.0, 49.0)	35.0 (27.5, 52.5)
Male, n (%)	109 (54.8%)	55 (55.6%)	54 (54.0%)
Ethnicity			
Caucasian/White	137 (68.8%)	70 (70.7%)	67 (67.0%)
Aboriginal and/or Torres Strait Islander	4 (2.0%)	1 (1.0%)	3 (3.0%)
Black	3 (1.5%)	1 (1.0%)	2 (2.0%)
Asian	25 (12.6%)	10 (10.1%)	15 (15.0%)
Other	30 (15.1%)	17 (17.2%)	13 (13.0%)
Travel overseas in the last 30 days, n (%)	5 (2.5%)	2 (2.0%)	3 (3.0%)
Contact with person known to be COVID infected, n (%)	134 (67.3%)	70 (70.7%)	64 (64.0%)
Health Care Worker, n (%)	35 (17.6%)	14 (14.1%)	21 (21.0%)
No risk factors, n (%)	8 (4.0%)	4 (4.0%)	4 (4.0%)
Unknown, n (%)	20 (10.1%)	11 (11.1%)	9 (9.0%)
WHO category*			
Not hospitalised with resumption of normal activities, n (%)	66 (33.3%)	29 (29.3%)	37 (37.4%)
Not hospitalised, but unable to resume normal activities, n (%)	131 (66.2%)	70 (70.7%)	61 (61.6%)
Hospitalised, requiring supplemental oxygen, n (%)	1 (0.5%)	0 (0.0%)	1 (1.0%)
Geometric mean viral load in copies/mL (95% CI)	537396 (323236, 893448)	700166 (332745, 1473299)^#^	424121 (208564, 862464)^

**Notes:** IQR, interquartile range; WHO, World Health Organisation; CI, confidence interval; * n=1 missing from placebo group; ^#^n=86; ^n=85.

**Figure 2:**
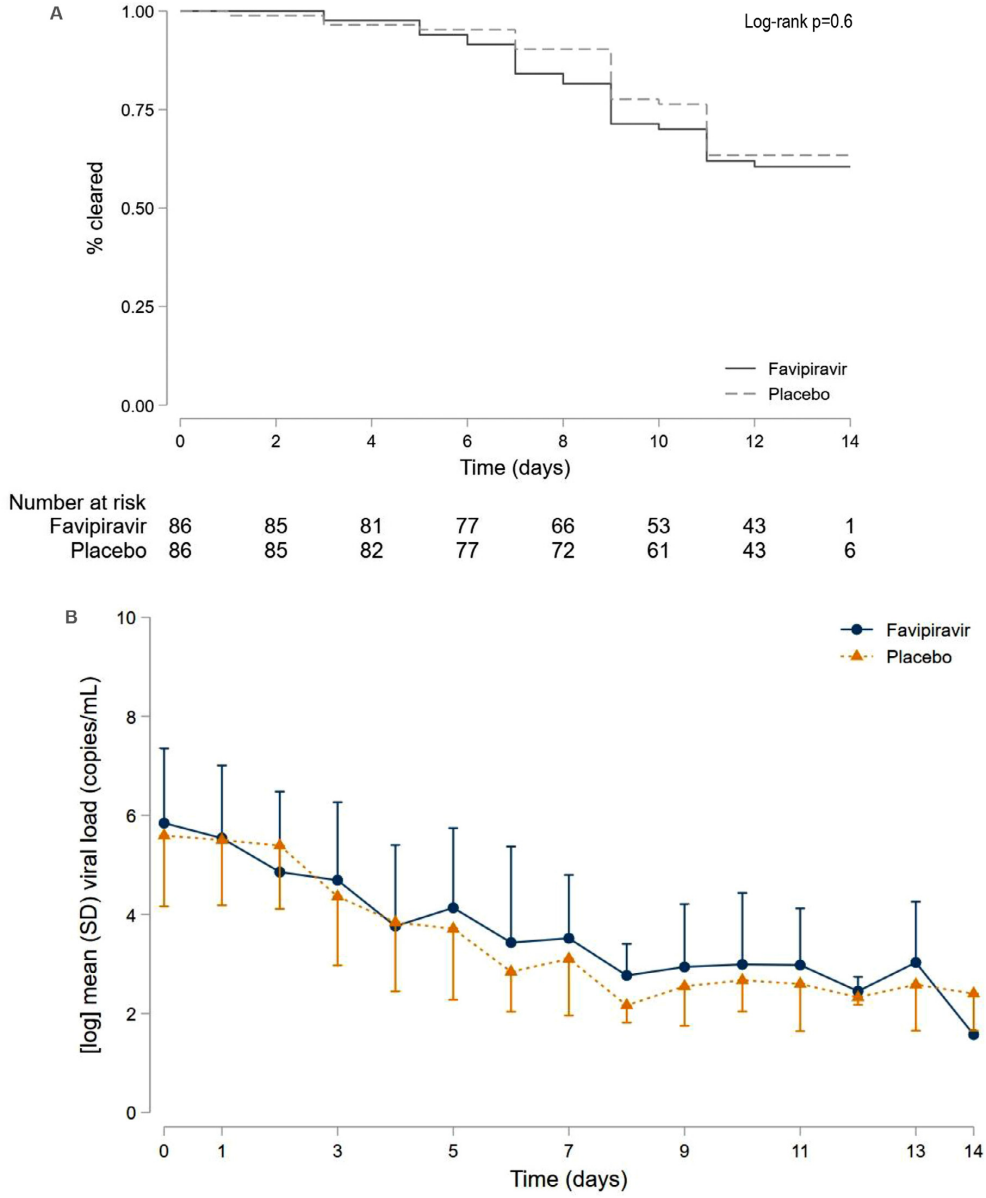
**Time to virological cure and SARS-CoV-2 viral load over time**. **(A) Time to virological cure.** Kaplan-Meier curves for 172 participants with evaluable data for time to 2 successive throat (or combined nose/throat) swabs negative for SARS-CoV-2 by nucleic acid testing. 60 participants met criteria for viral clearance. **B) Mean SARS-CoV-2 viral load in log_10_ copies/mL over 14 days post enrolment.** There was no difference in mean log_10_ viral load at day 5 (*p*=0.26) or day 13 (*p*=0.13) and no difference in change in viral load from baseline to day 5 whether adjusted (*p*=0.32) or unadjusted (*p*=0.57) for baseline viral load. Error bars represent half the standard deviation (SD).

There was no difference in the primary endpoint of time to virological cure between favipiravir and placebo treatment groups (Log-rank *p*=0.6) and no difference in viral load over the 14 days of dosing (Figure 2). This analysis included 172 participants with at least one assessment of SARS-CoV-2 viral load and excluded results where there was no detectable internal control (RPP30) and SARS-CoV-2 RNA. Sensitivity analyses did not identify any differences in time to virological cure between the treatment arms (Figure S1).

In the ITT population, 66 (33.2%) were WHO category I, 131 (65.8%) were WHO category 2 and 1 (0.1%) participant was WHO category 4 at baseline. Progression to hospitalisation for those in the community (WHO category 1 or 2) occurred in 14 people receiving favipiravir and 9 receiving placebo (*p*=0.38). Progression to the need for oxygen supplementation (WHO category 4 or 5) occurred in 6 people in each of the study arms (*p*=1.0). Similar results were seen in the mITT (*p*=0.37) and the PP populations (*p*=0.10) for progression to hospitalisation and for progression to oxygenation (both *p*=1.0). Clinical progression in the PP population was lower than the ITT population as people who were admitted were less likely to complete all doses of study medication. No trial participants died or required invasive mechanical ventilation.

**Figure 3:**
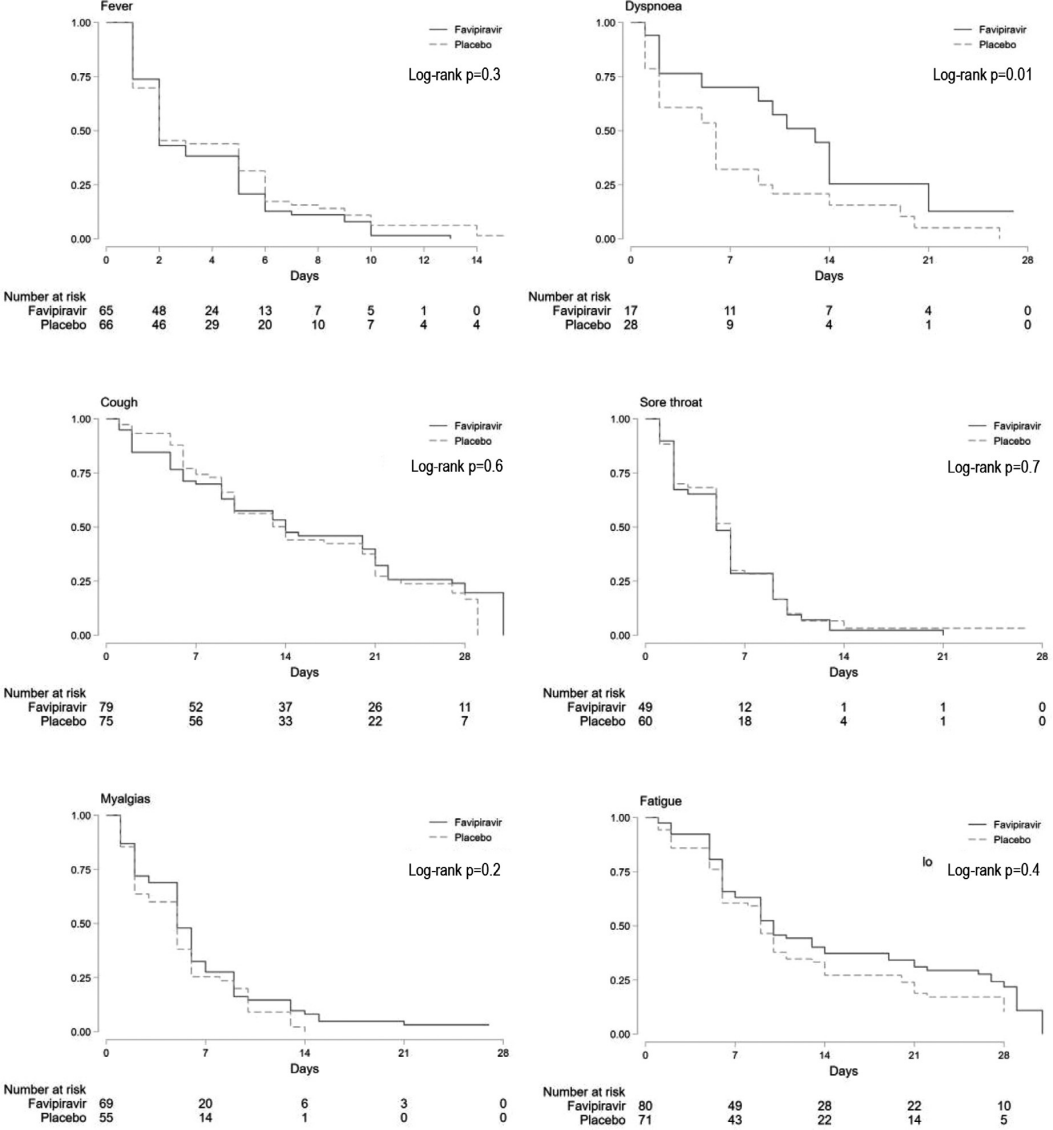
**Time to symptom resolution**. Kaplan-Meier curves for participants in the mITT population who reported fever, dyspnoea, cough, sore throat, myalgia and fatigue at enrollment.

There were no significant differences in time to resolution of fever, cough or sore throat in both the mITT and PP populations when these symptoms were reported at baseline (Figure 3). Dyspnoea resolved more quickly in people receiving placebo (*p* = 0.01) and was also more common in that group (*n*=28) compared to favipiravir (*n*=17). In both groups, 75% of participants who reported dyspnoea at baseline had resolution by day 14. Similar results were seen in the PP population (Figure S2).

Adverse events were reported in 67.4% of participants in the mITT population with 26.8% of participants having events that were possibly related to study drug. Among the 128 (67.4%) participants reporting at least 1 adverse event, there were a total of 331 events, 176 in the favipiravir arm and 155 in the placebo arm, with 48 (27.3%) and 41 (26.5%) of events possibly related to study drug, respectively (Table 2). The most common related adverse events were gastrointestinal events such as nausea, vomiting and diarrhoea (Table S1). The only serious adverse events were hospitalisations which were all considered unrelated to study drug and no individuals died. Laboratory data were available at baseline and 2 weeks after completing study drug for the first 82 enrolled individuals. (Table S2). There were no clinically significant differences by study arm in these data, so a protocol amendment was approved for ongoing enrolment without routine laboratory monitoring.

**Table 2:** Summary of adverse events (AEs) for mITT population. Adverse events were any symptoms that developed after enrolment, related AEs were considered possibly related to study drug.

	Overall (*n*=190)	Favipiravir (*n*=95)	Placebo (*n*=95)
**Participants reporting Adverse Events N (%)**	128 (67.4%)	63 (49.2%)	65 (50.8%)
**Adverse Events, N**	331	176	155
**Participants reporting related Adverse Events, N (%)**	51 (26.8%)	24 (47.1%)	27 (52.9%)
**Related Adverse Events, N**	89	48	41
**Serious Adverse Events,**^1^**N**	23	14	9
**Deaths, N**	0	0	0

1 Notes: All Serious adverse events were hospitalisations, and none were considered related to study drug.

## Discussion

The data presented here demonstrate that favipiravir does not improve virologic or clinical outcomes in early symptomatic COVID-19. Favipiravir showed no benefit over placebo for the primary endpoint of viral clearance and the secondary endpoints of progression in clinical disease and symptom resolution. In addition, multiple sensitivity analyses for alternative definitions of viral clearance demonstrated no benefit of favipiravir and we conclude that no antiviral effect was evident in this trial.

Antivirals are likely to have the highest benefit if administered early in the course of COVID-19 and placebo controlled trials will provide the highest level evidence if such a benefit exists.^4^, ^5^, ^6^ Favipiravir has generated interest as a therapy for COVID-19 due to its mechanism of action by inhibiting viral replication and its potential for oral dosing at pharmacokinetic parameters that confer virologic activity.^7^^,^^8^ There have been multiple open label studies of favipiravir in hospitalised people later in the course of disease with mixed findings, including trials from China which reported faster clinical recovery in moderately unwell participants,^10^ a trial of 500 hospitalised people in Malaysia which reported no benefit in preventing clinical progression,^14^ an Indian trial of 150 hospitalised people which reported faster clinical recovery but no difference in time to PCR negativity^15^ and 2 smaller trials with under 100 participants which each reported improvement in fever resolution with one of the trials also reporting reduced time to PCR negativity.^16^

These trials are included in the over 110 clinical trials of favipiravir for COVID-19,^17^ yet only two have specifically studied early disease with a placebo controlled design, both also reporting no benefit with favipiravir.^18^^,^^19^ The trial of 231 people from Saudi Arabia used a short 5 day course of favipiravir or placebo and excluded people with major medical co-morbidities and allowed enrolment of people with mild illness only, including those with gastrointestinal symptoms as their manifestation of COVID-19.^18^ The second trial from the United States enrolled 149 people but only assessed virologic outcomes in a subset of 116 people. In contrast to our study some participants were asymptomatic and had been vaccinated at trial entry.^19^ In addition neither of these studies examined SARS-CoV-2 viral load or had clinical assessments every 2 days during early infection. These data combined with results of our trial with repeated assessments of SARS-CoV-2 viral load while receiving favipiravir or placebo support the lack of virological or clinical benefit of favipiravir as a treatment for early symptomatic COVID-19.

This study also highlights the challenges of pharmacokinetics and dosing of repurposed drugs for a new viral pathogen. Prior to trial commencement published data supported *in vitro* activity with a half maximal effective concentration (EC50) of 62 μM (10 μg/ml)^8^ that is above the trough levels reported in healthy volunteers and those in influenza clinical trials at the same or lower dosing used in our trial.^12^^,^^20^ This combined with evidence from early open label studies in China of clinical benefit at this same dosing.^9^ Subsequent data have suggested higher dosing as when trialled for Ebola Virus infection (6000 mg on day 0 followed by 1200 mg BID for 9 days) generate trough levels more comparable to effective doses in an animal model of SARS-CoV-2 infection.^21^^,^^22^ and EC50s of 118 μM (19 μg/ml) in other *in vitro* models.^23^ While multiple *in vitro* and *in vivo* animal pharmacokinetic models to provide the best rationale for dosing would be preferable, the nature of a rapidly evolving pandemic means multiple pre-clinical evaluations are not necessarily available at the time of selecting candidates for clinical trials. Result of our trial highlights the importance of rapid evaluation of treatment candidates in multiple pre-clinical models to assist selection of therapeutic candidates for an emerging viral pathogen

Favipiravir has been authorised as a treatment for COVID-19 in countries such as Japan, Russia, Serbia, Turkey, India, and Thailand, under emergency provisions and is still available in many of these countries in generic formulations. The COVID-19 pandemic has seen the widespread use of many therapeutics where no benefit has been demonstrated and before clinical trials have demonstrated improved clinical outcomes.^24^, ^25^, ^26^ This trial demonstrates the importance of performing controlled studies with clinical and virological endpoints to inform clinicians and COVID-19 programmes about potential treatments. Our trial provides a model for smaller clinical trials that can potentially avoid the need to rapidly move into large phase 3 trials that repurpose existing medications based on activity against SARS-CoV-2 from pre-clinical data.

Despite the strengths of our design the study has some limitations. Firstly, it was conducted in an era before Omicron and related lineage variants. However other directly acting antivirals have maintained activity in the setting of Omicron and related variants^27^ so our data should still be valid in the current clinical environment. In addition, our study may have been underpowered to predict clinical progression as we did not focus on enrolling people with risk factors for poor clinical outcomes. However, this trial focussed on identifying an antiviral effect of favipiravir in addition to evidence of clinical benefit with frequent sampling and clinical review. Importantly data from licensing studies of drugs used to treat early COVID-19 such as nirmatrelvir/ritonavir, molnupiravir and sotrovimab have demonstrated an antiviral effect reporting greater reductions in SARS-CoV-2 viral load with drug compared to placebo on serial nose-throat swabs.^4^^,^^6^^,^^28^ Remdesivir also has reports of reductions in viral load although not in the licensing study for early disease.^5^^,^^29^^,^^30^ Our findings demonstrating no antiviral effect or clinical benefit of favipiravir with a placebo controlled design are consistent with findings from these trials of therapies conferring benefit. It also highlights a trial design that provides high quality clinical and virological evidence that larger trials with favipiravir are not required in this patient population. Another limitation of our study is we were unable to conduct formal medication reconciliation or pill counts to confirm study adherence. Adherence was emphasized through the second daily calls to participants but due to efforts to minimise contact between participants with infectious COVID-19 and study staff this reconciliation was not performed.

In conclusion, our data do not support the use of favipiravir in early symptomatic COVID-19 infection and jurisdictions where favipiravir is currently available should consider reviewing its access to treat COVID-19.

## Contributors

JHM and AYP conceptualized and designed the study. SJL, JHM and AYP analysed and interpreted the data and drafted the figures and tables. JHM JSYL, BR, AC, SJL and AYP drafted the protocol. JHM acquired the funding. AC, JR, MB, SP, JG, MH, JO'B, AP collected and entered the data and administered the project. AH and GT designed and performed the virological analyses. LB, JAW, SB, GZB, SP ad KP collected and processed all study specimens. HP, SG, NP, CP, RM, BR, JT, KC, MK, JM, AP, BG and JSYL identified study participants for enrolment. JHM and JSYL consented participants, performed study procedures and assessments of adverse effects. JHM wrote the first draft of the manuscript. All authors contributed, reviewed and edited the manuscript.

## Data sharing statement

Anonymized raw data will be available upon a written request to the corresponding author. The data will be shared with requesters after approval and a signed data-sharing agreement between the requester and the sponsor Alfred Health.

## Declaration of interests

All authors declare no competing interests.

## Appendix Supplementary materials

Image, application 1
