# Supplementary material for: Favipiravir in early symptomatic COVID-19, a randomised placebo-controlled trial
Source: eClinicalMedicine. 2022 Oct 20;54:101703. doi: 10.1016/j.eclinm.2022.101703 (PMC9583769; doi:10.1016/j.eclinm.2022.101703)
Supplement: Supplementary file 1 [file mmc1.docx]

**Supplementary Tables and Figures**

**Table S1. Clinical adverse events (AEs) for mITT population.** Adverse events are any symptoms that develop after enrolment, related AEs were considered possibly related to study drug

|  | **Overall**  **(n=190)** | **Favipiravir (n=95)** | | **Placebo (n=95)** | |
| --- | --- | --- | --- | --- | --- |
|  | **All AEs** | **All AEs** | **Related AEs** | **All AEs** | **Related AEs** |
| **Symptom** | **N (%)** | **N (%)** | **n** | **N (%)** | **n** |
| Fever | 13 (6.8%) | 11 (84.6%) | 1 | 2 (15.4%) | 0 |
| Dyspnoea | 23 (12.1%) | 11 (47.8%) | 1 | 12 (52.2%) | 0 |
| Cough | 9 (4.7%) | 6 (66.7%) | 0 | 3 (33.3%) | 0 |
| Sore throat | 6 (3.2%) | 3 (50%) | 0 | 3 (50%) | 0 |
| Rhinorrhoea | 16 (8.4%) | 5 (31.3%) | 1 | 11 (68.8%) | 0 |
| Headaches | 10 (5.3%) | 4 (40%) | 0 | 6 (60%) | 1 |
| Myalgias | 5 (2.6%) | 3 (60%) | 0 | 2 (40%) | 0 |
| Diarrhoea | 23 (12.1%) | 12 (52.2%) | 11 | 11 (47.8%) | 10 |
| Fatigue | 8 (4.2%) | 5 (62.5%) | 0 | 3 (37.5%) | 0 |
| Loss of smell | 38 (20%) | 23 (60.5%) | 3 | 15 (39.5%) | 2 |
| Loss of taste | 34 (17.9%) | 18 (52.9%) | 2 | 16 (47.1%) | 3 |
| Skin changes | 16 (8.4%) | 7 (43.8%) | 4 | 9 (56.3%) | 4 |
| Vomiting | 7 (3.7%) | 3 (42.9%) | 3 | 4 (57.1%) | 3 |
| Nausea | 18 (9.5%) | 10 (55.6%) | 9 | 8 (44.4%) | 7 |
| Nasal congestion | 21 (11.1%) | 10 (47.6%) | 0 | 11 (52.4%) | 0 |
| Cardiac other | 3 (1.6%) | 1 (33.3%) | 0 | 2 (66.7%) | 0 |
| Neurological other | 15 (7.9%) | 6 (40%) | 0 | 9 (60%) | 3 |
| Muscular skeletal other | 22 (11.6%) | 12 (54.5%) | 2 | 10 (45.5%) | 1 |
| Psychological other | 3 (1.6%) | 1 (33.3%) | 0 | 2 (66.7%) | 0 |
| Swollen lymph nodes | 7 (3.7%) | 6 (85.7%) | 0 | 1 (14.3%) | 0 |
| Mucositis other | 6 (3.2%) | 5 (83.3%) | 1 | 1 (16.7%) | 0 |
| Urogenital other | 1 (0.5%) | 0% | 0 | 1 (100%) | 0 |
| Gastrointestinal other | 22 (11.6%) | 12 (54.5%) | 9 | 10 (45.5%) | 7 |
| Pain | 2 (1.1%) | 0% | 0 | 2 (100%) | 0 |
| Confusion/delirium | 1 (0.5%) | 1 (100%) | 0 | 0% | 0 |
| Other unclassified | 2 (1.1%) | 1 (50%) | 1 | 1 (50%) | 0 |

**Table S2. (A) Summary estimates of laboratory data at Day 0 and Day 28 for first 82 enrolled participants**

|  | **Baseline median (IQR)** | | | | **Day 28 median (IQR)** | | | | **Median change (IQR) =day28-day0** | | | |
| --- | --- | --- | --- | --- | --- | --- | --- | --- | --- | --- | --- | --- |
|  | **n** | **Placebo** | **n** | **Favipiravir** | **n** | **Placebo** | **n** | **Favipiravir** | **n** | **Placebo** | **n** | **Favipiravir** |
| **WBC (x 10^9^ cells per litre)** | 39 | 4.96 (3.75, 6.06) | 43 | 4.54 (3.61, 5.84) | 35 | 5.86 (5.07, 7.33) | 31 | 5.65 (5.15, 6.64) | 35 | 0.78 (-0.18, 1.47) | 30 | 1.58 (0.20, 2.07) |
| **Lymphocytes (x 10^9^ cells per litre)** | 39 | 1.53 (1.23, 1.89) | 43 | 1.55 (1.17, 1.88) | 35 | 1.97 (1.64, 2.37) | 31 | 1.80 (1.58, 2.46) | 35 | 0.27 (0.02, 0.60) | 30 | 0.37 (0.06, 0.71) |
| **Neutrophils (x 10^9^ cells per litre)** | 39 | 2.69 (1.70, 3.44) | 43 | 2.19 (1.73, 3.33) | 35 | 3.49 (2.57, 4.14) | 31 | 3.19 (2.39, 3.56) | 35 | 0.46 (-0.58, 1.21) | 30 | 0.77 (-0.03, 1.44) |
| **Hb (g/L)** | 39 | 149 (134, 159) | 43 | 146 (139, 152) | 35 | 139 (126, 147) | 31 | 140 (133, 146) | 35 | -8 (-12, -3) | 30 | -6.5 (-12, -5) |
| **Platelets (x 10^9^ cells per litre)** | 39 | 209 (167, 249) | 43 | 214 (174, 243) | 35 | 227 (192, 284) | 31 | 246 (231, 294) | 35 | 13 (2, 43) | 30 | 29 (14, 57) |
| **Bilirubin (μmol/L)** | 38 | 8.5 (6, 12) | 42 | 7 (5, 10) | 34 | 10 (8, 14) | 31 | 10 (7, 13) | 32 | 2.5 (-1, 5) | 30 | 2.5 (1, 5) |
| **ALT (IU/L)** | 39 | 31 (18, 66) | 43 | 25 (17, 39) | 33 | 27 (17, 41) | 31 | 27 (20, 69) | 32 | -2 (-11, 1) | 30 | 1 (-4, 10) |
| **Albumin (g/L)** | 40 | 42 (39, 44) | 43 | 42 (39, 44) | 34 | 41 (39, 44) | 31 | 41 (38, 44) | 34 | -1 (-3, 0) | 30 | 0 (-1, 1) |
| **Creatinine (μmol/L)** | 40 | 74 (61, 85) | 43 | 73 (64, 84) | 34 | 68.5 (59, 78) | 31 | 68 (64, 75) | 34 | -5 (-10, 0) | 30 | 0 (-6, 3) |

**(B) Graded laboratory adverse events where there was an increase in grade from baseline to day 28 (n=82).** Reported for haemoglobin, white blood cell count, lymphocytes, platelets, bilirubin, ALT, albumin and creatinine. Includes relevant clinical data. Grading according to Division of AIDS (DAIDS) Table for Grading the Severity of Adult and Pediatric Adverse Events, Version 2.1 (July 2017)

| **Event** | **VIRCO ID** | **Sex** | **Baseline Grade** | **Day 28 Grade** | **Baseline value** | **Day 28 value** | **Selected Clinical Data** | **Study Arm** |
| --- | --- | --- | --- | --- | --- | --- | --- | --- |
| Bilirubin (μmol/L) high | VALC027 | Female | Normal | Grade 1 | 11 | 27 | No past history. Recovered apart from loss of smell | Favipiravir |
|  | VALC012 | Male | Normal | Grade 2 | 15 | 38 | No past history. Recovered apart mild cough and slight rash on toes | Placebo |
| ALT (IU/L) high | VALC008 | Male | Normal | Grade 2 | 17 | 126 | No past history. Recovered | Favipiravir |
|  | VALC021 | Male | Normal | Grade 1 | 34 | 75 | Hyperlipidaemia on atorvastatin at baseline. Recovered | Favipiravir |
|  | VALC023 | Male | Normal | Grade 1 | 33 | 69 | No past history. Recovered | Favipiravir |
|  | VALC038 | Female | Normal | Grade 1 | 35 | 45 | On oral cephalexin at baseline. Admitted Day 13 with fever / cough / hepatitis (ALT peak of 339 IU/L) / thrombocytopenia (down to 67 x 10^9^/litre). Events related to COVID | Favipiravir |
|  | VALC061 | Male | Grade 1 | Grade 2 | 90 | 145 | No past history. Recovered apart from mild cough. Receiving regular Panadol up until study day 7 | Placebo |
|  | VALC084 | Male | Grade 1 | Grade 2 | 55 | 112 | Smoker, Hyperlipidaemia on atorvastatin at baseline. Recovered | Favipiravir |

**Figure S1. Time to virological cure: sensitivity analyses.** Kaplan-Meier curves for time to 2 successive throat (or combined nose/throat) swabs negative for SARS-CoV-2 by nucleic acid testing.

**A) Consider SARS-CoV-2 RNA viral load < 300 copies/mL as a negative viral load result.** 169 participants included in analysis and 114 meet criteria for viral clearance

**B) Diregarding results for the control gene and analysing based on results of SARS-CoV-2 RNA only**. 173 participants included in analysis and 82 meet criteria for viral clearance

**C) Disregarding results if the control gene was undetectable regardless of the results for SARS-CoV-2 RNA.** 170 participants included in analysis and 60 meet criteria for viral clearance

**D) Defining viral clearance based on a single SARS-CoV-2 viral load result being undetectable**. Uses same population as for the study primary outcome. 172 participants included in analysis and 104 met criteria for viral clearance

**Figure S2. Time to symptom resolution.** Kaplan-Meier curves for participants in the PP population who reported fever, dyspnoea, cough, sore throat , fatigue and myalgia an enrollment.
